# Corosolic acid inhibits the proliferation of glomerular mesangial cells and protects against diabetic renal damage

**DOI:** 10.1038/srep26854

**Published:** 2016-05-27

**Authors:** Xiao-Qiang Li, Wen Tian, Xiao-Xiao Liu, Kai Zhang, Jun-Cheng Huo, Wen-Juan Liu, Ping Li, Xiong Xiao, Ming-Gao Zhao, Wei Cao

**Affiliations:** 1 Department of Pharmacology, School of Pharmacy, Fourth Military Medical University, Xi’an 710032, China; 2 Cadet Brigade, Fourth Military Medical University, Xi’an 710032, China; 3 Department of Natural Medicine & Institute of Materia Medica, School of Pharmacy, Fourth Military Medical University, Xi’an 710032, China

## Abstract

Diabetic nephropathy (DN) is one of the major complications of diabetes mellitus (DM). This study aimed to explore the effects of corosolic acid (CA) on the renal damage of DM and the mechanisms behind these effects. The renoprotective effect of CA was investigated in type 1 diabetic rats and *db/db* mice. The kidneys and glomerular mesangial cells (GMCs) were used to study the proliferation of GMCs by immunostaining and MTT assay. Further immunoblotting, siRNA, qPCR analysis, and detecting of NADPH oxidase activity and reactive oxygen species (ROS) generation were performed to explore relevant molecular mechanisms. In CA-treated diabetic animals, diabetes-induced albuminuria, increased serum creatinine and blood urea nitrogen were significantly attenuated, and glomerular hypertrophy, mesangial expansion and fibrosis were ameliorated. Furthermore, CA significantly inhibited proliferation of GMCs and phosphorylation of ERK1/2 and p38 MAPK in both diabetic animals and high glucose (HG)-induced GMCs. CA also normalized Δψm and inhibited HG-induced NADPH oxidase activity, ROS generation and NOX4, NOX2, p22^phox^ and p47^phox^ expression. More importantly, CA inhibited GMC proliferation mediated by NADPH/ERK1/2 and p38 MAPK signaling pathways. These findings suggest that CA exert the protective effect on DN by anti-proliferation resulted from inhibition of p38 MAPK- and NADPH-mediated inactivation of ERK1/2.

Diabetic nephropathy (DN), also known as diabetic glomerulosclerosis, is increasingly recognized as a major complication of diabetes mellitus (DM)^1^. DN is the single most common cause of end-stage renal disease (ESRD) in adults, and is characterized by a series of renal structural changes including mesangial expansion, glomerular basement membrane thickening, glomerulosclerosis, and in advanced stages, tubulointerstitial fibrosis^2^,^3^. Until now, glycemic control, blood pressure control, and inhibition of the renin-angiotensin-aldosterone system have been shown to slow the progression of DN^4^. However, the role of optimizing control to retard, prevent or reverse DN remains controversial^5^, and the number of patients with DN that ultimately develop ESRD remains unacceptably high^4^. The treatment of DN is therefore still an unresolved issue posing a formidable challenge^3^,^6^.

Corosolic acid (2α-hydroxyursolic acid, CA), a pentacyclic triterpenoid isolated from *Lagerstroemia speciosa* L^7^, exhibits anti-tumor^8^, anti-diabetic^9^, anti-obesity^10^, and anti-inflammatory activities^11^. An increase in cellular uptake of glucose and induction of apoptosis by this compound have been suggested to explain its beneficial effects. CA also modulates a wide array of signal transduction pathways, including signal transducer and activator transcription-3, NF-κB, protein kinase C, β-catenin, procaspase-3, -8, and -9, Fas and AMP-activated protein kinase^12^,^13^,^14^,^15^,^16^. Despite the numerous pharmacological activities identified for CA, there is little data available regarding its effect on DN. In the current study, the therapeutic value of CA on DN was evaluated both in streptozotocin (STZ)-induced diabetic rats and diabetic *db/db* mice. We found that CA could markedly ameliorate renal injury *in vivo*, characterized as decreasing levels of urinary albuminuria, blood urea nitrogen (BUN), and serum creatinine in diabetic animals, further inhibiting mesangial expansion.

Glomerular mesangial cells (GMCs), one of the major constituents of the renal glomerulus, play important roles in mesangial matrix homeostasis, regulation of glomerular filtration rate, and phagocytosis of apoptotic cells in the glomerulus^17^. Hyperglycemia is a crucial risk factor in the development of DN because of its alteration of glomerular mesangial matrix clustering as well as irregularity and widening of glomerular capillaries, thickening in foci of glomerular basement membrane, and marked increase in GMC proliferation and hypertrophy^18^,^19^. Under diabetic conditions, high blood glucose levels are responsible for the proliferation of GMCs *in vitro*^4^. Several mechanisms have been considered to be involved in the proliferation of GMCs, including activation of PKC-mitogen-activated protein kinases (MAPK), increased oxidative stress, renal polyol formation, and accumulation of advanced glycation end products. There are four different MAPK cascades: the extracellular signal-regulated kinases (ERK1/2), c-Jun N-terminal kinases (JNK), p38MAPK, and extracellular signal-regulated kinase-5 (ERK5/BMK1)^20^. Studies have shown that MAPKs are upregulated in the glomeruli of diabetic animals, and attenuation of the MAPK pathway in the renal cortex could be one beneficial effect of intensive insulin therapy^21^. ERKs and p38 are preferentially phosphorylated (activated) in GMCs exposed to high glucose (HG) conditions and regulate cell proliferation, differentiation and survival^22^,^23^.

Further, since the first demonstration of NOX (NADPH oxidase) enzyme expression in the kidney in the early 1990s, accumulated evidence has indicated that the NOX family of nicotinamide adenine dinucleotide phosphate (NADPH) reduced form oxidases, as a major source of reactive oxygen species (ROS), contributes significantly to the initiation and development of DN^24^,^25^. To date, the NOX family comprises seven members: NOX1-5 and the dual oxidases (Duox)-1 and -2 (Renox)^26^. NOX4 and NOX2 are the NOX homologues that are predominantly expressed in GMCs of humans, mice and rats. In response to HG, NOX4 interacts with the p22^phox^ subunit and enhances its activity^27^, whereas NOX2 binds to p47^phox^, as an activating, stabilizing and/or regulatory subunit. Downregulation of NOX blunts HG-induced ROS and extracellular matrix accumulation^26^.

Therefore, in order to understand the cellular events that CA mediates to inhibit mesangial expansion in DN, we examined the effect of CA on GMCs and related cell signaling. We found that CA downregulated MAPK activation, including phosphorylation of p38 MAPK and ERK1/2, leading to dose-dependent inhibition of proliferation of GMCs. In addition, we provide the first evidence that CA blocks expression of NOX4, NOX2, and NOX-associated subunits p22^phox^ and p47^phox^, inhibiting activation of ERK1/2 signal transduction, resulting in inhibition of GMC proliferation and thereby attenuating mesangial expansion in response to HG. These results suggest that CA exert beneficial effects on DN, and represent a novel mechanism of CA-mediated inhibition of HG-induced GMC proliferation.

## Results

### Effects of CA on blood glucose of diabetic animals

Blood glucose levels of the experimental rats were measured at weeks 0, 2, 4 6 and 8 after treatment with CA. Compared with the age-matched normal rats, STZ-treated rats had higher glucose levels throughout the treatment process (Table 1). On week 8, STZ-treated rats exhibited hyperglycemia, with blood glucose (26.16 ± 1.42 mmol/L) being significantly higher than control rats (6.48 ± 0.38 mmol/L, *P* < 0.05). CA treatment markedly attenuated hyperglycemia in diabetic rats, and the effect appeared to be dose-dependent, whereas the blood glucose value in the CA-treated normal group was similar to the value in normal rats throughout the treatment process.

The effect of CA on the blood glucose of *db*/*db* mice are shown in Supplementary Fig. S1, the blood glucose were markedly increased in *db*/*db* mice compared with non-diabetic mice but were significantly improved with CA treatment.

### Effects of CA on renal dysfunction of diabetic animals

In order to confirm the mimicking of type 1 diabetes by STZ injection in rats and the resulting consequences on the renal system, we monitored the kidney index and renal functional parameters including urinary albuminuria, BUN, and Serum creatinine (Cre). Kidney enlargement as assessed by an increased kidney index was found in the diabetic group, but was significantly reduced by treatment with both CA and enalapril (Table 2). Furthermore, the levels of Cre, BUN and urinary albuminuria were significantly higher in model rats than control. CA treatment displayed a dose-dependent decrease in these renal functional parameters in diabetic rats, and the decrease in the 20 mg/kg CA-treated diabetic group was even more pronounced than in the enalapril-treated group.

**Figure 1:**
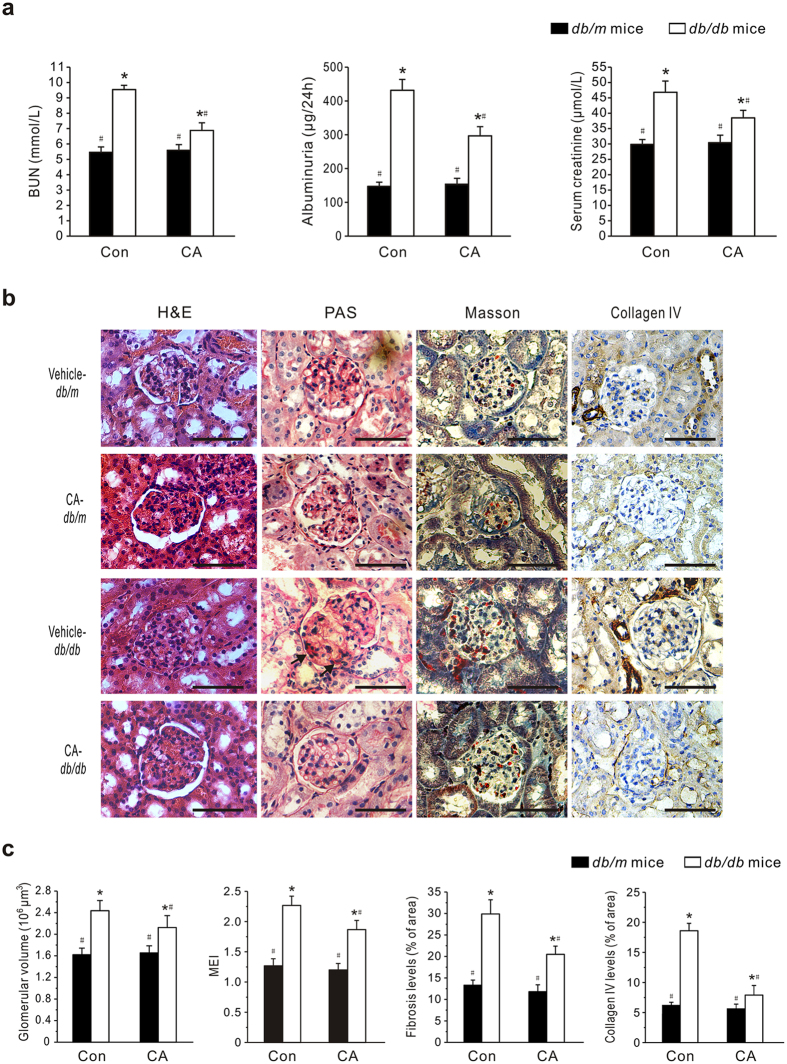
The effects of corosolic acid (CA) on renal function of *db/db* mice. Eight-week-old *db/db* and age-matched *db/m* mice (Con) were treated with CA (10 mg/kg/day) or vehicle (0.5% CMC) by gavage for 8 weeks. (**a**) Blood urea nitrogen (BUN), Urinary albuminuria and serum creatinine were measured using commercial assay kits. (**b**) Representative photomicrographs of the kidneys stained with H&E, periodic acid-Schiff (PAS), Masson’s and collagen IV immunohistochemical staining (original magnification 400×). Scale bars: 100 μm. Arrows indicate mesangial matrix expansion. (**c**) Quantitative assessments of glomerular volume, glomerular mesangial expansion, glomerular fibrosis and glomerular collagen IV expression. Data are presented as mean ± SEM (n = 30). *P < 0.05 *vs*. vehicle-treated *db/m* mice; ^#^*P* < 0.05 *vs*. vehicle-treated *db/db* mice.

Furthermore, urinary albuminuria, BUN and Cre were markedly increased in *db*/*db* mice compared with non-diabetic mice but were significantly improved with CA treatment (Fig. 1a).

### Effects of CA on renal structure

The renoprotective effect of CA was also assessed by histopathological analysis. Glomerular structures were examined by H&E, periodic acid-Schiff (PAS), Masson’s stains and collagen IV immunohistochemical stains. As shown in Fig. 1b, kidneys from vehicle-treated *db*/*db* mice revealed obvious swelling and denaturation of glomeruli. In some cases, the lumen of the tubules in *db*/*db* mice was unnaturally widened, the epithelial cells were severely damaged, and the tubular basement membrane was broken and resembled bristles. Further examination of PAS and Masson’s-stained kidney tissue sections from the diabetic mice showed mesangial matrix expansion and fibrosis. Meanwhile, the expression of collagen IV was up-regulated in the diabetic mice. After the 8-week treatment with CA, glomerular hypertrophy, mesangial accumulation, fibrosis and collagen development in the *db*/*db* mice were significantly inhibited. In addition, there were no notable changes in renal histology in CA-treated *db*/*m* mice. According to the statistical assessment of the results (Fig. 1c), the glomerular volume and mesangial expansion, assessed by the mesangial expansion index (MEI), was significantly larger in untreated *db/db* mice than in *db/m* mice, whereas CA treatment significantly attenuated glomerular volume and MEI increase in *db/db* mice (*P* < 0.05). Additionally, treatment of *db*/*db* mice with 10 mg/kg CA caused 31.1% reduction of glomerular fibrosis level and 66.7% reduction of collagen IV expression relative to model counterparts.

**Figure 2:**
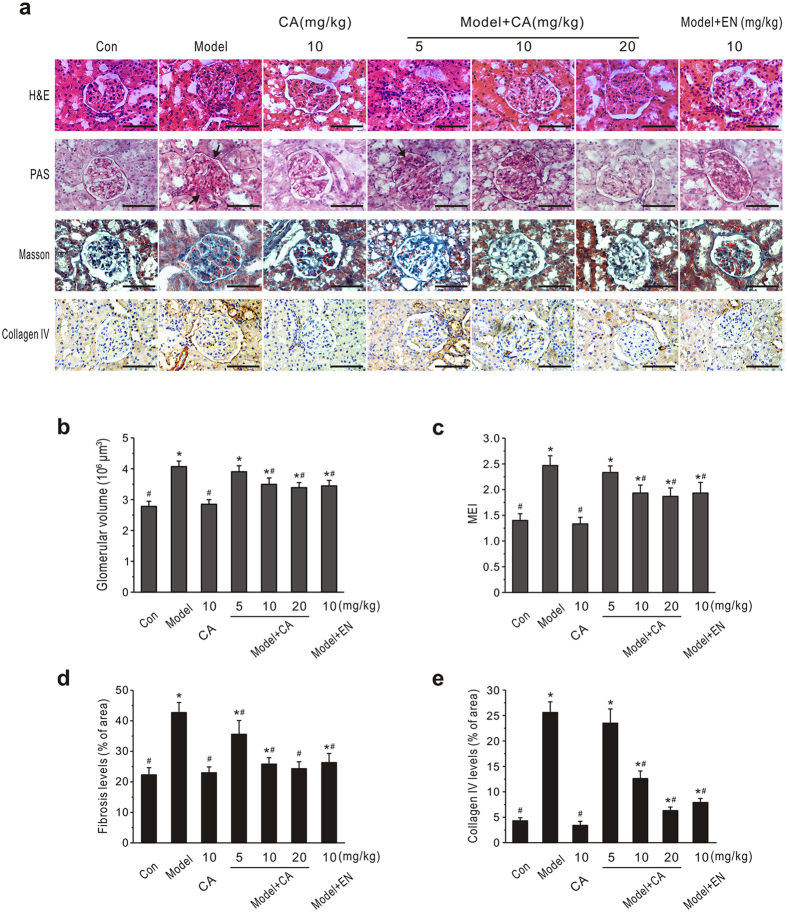
Effects of CA on renal pathology of diabetic rats. CA and enalapril (EN) were administered daily by gavage to diabetic (model) or normal (Con) rats for 8 weeks. (**a**) Representative photomicrographs of H&E, periodic acid-Schiff (PAS), Masson’s and collagen IV immunohistochemically stained rat kidneys. Arrows indicate the mesangial matrix expansion and increased thickness of the glomerular basement membrane. Scale bars: 100 μm. (**b**) Statistical analysis of glomerular volume. (**c–e**) Quantitative assessment of glomerular mesangial expansion, glomerular fibrosis and glomerular collagen IV expression. Data are presented as mean ± SEM (n = 30). *P < 0.05 *vs*. control; ^#^*P* < 0.05 *vs*. model.

Similarly, there was obvious hypertrophy, hyperplasia and fibrosis of the whole glomerulus in the STZ-induced diabetic group (Fig. 2a). Enalapril treatment significantly reduced the changes in glomerular structure in diabetic rats. Similar features were observed in kidney tissues from 10 or 20 mg/kg CA-treated diabetic groups. Statistical assessment showed that CA treatment dose-dependently decreased the glomerular volume in diabetic rats (Fig. 2b). Based on PAS-stained sections, diabetic rats displayed accelerated mesangial matrix expansion and increased thickness of the glomerular basement membrane. The MEI of normal rats was 1.4 ± 0.13, while it markedly increased to 2.47 ± 0.19 in the diabetic rats (*P* < 0.05, Fig. 2c). The MEI values for CA- and enalapril-treated diabetic groups were significantly reduced, and CA reduced this index in a dose-dependent manner. CA treatment, particularly at 20 mg/kg, inhibited the glomerular fibrosis and down-regulated collagen IV expression significantly (*P* < 0.05, Fig. 2d,e).

**Figure 3:**
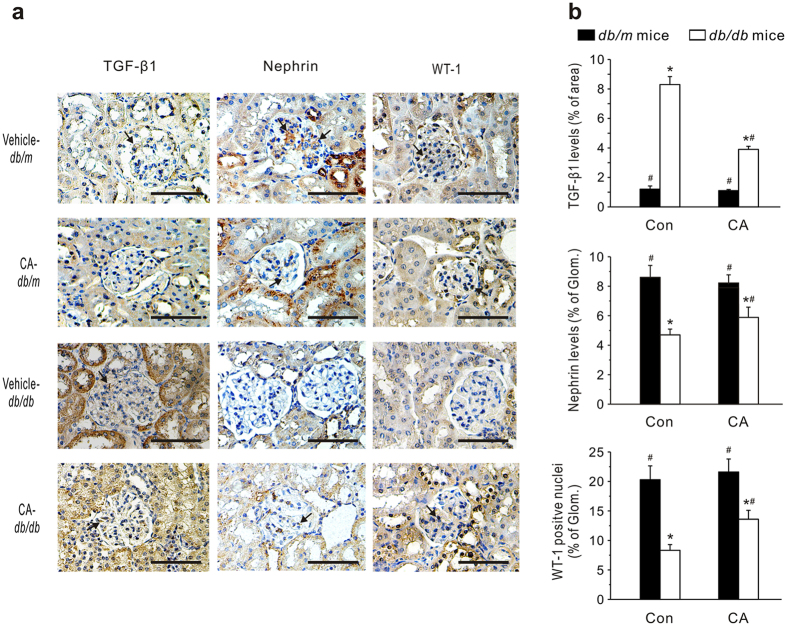
Effects of CA on glomerular TGF-β1, nephrin and Wilms’ tumor-1 (WT-1) levels in *db/db* mice. (**a**) Representative immunohistochemical images. Scale bars: 100 μm. (**b**) Quantitative assessments of immunohistochemical staining in the kidneys. Twenty glomeruli were randomly selected from four kidneys in each group. Data are presented as mean ± SEM *P < 0.05 *vs*. vehicle-treated *db/m* mice; ^#^*P* < 0.05 *vs*. vehicle-treated *db/db* mice.

Furthermore, we immunohistochemically stained the kidney using antibodies against transforming growth factor-β1 (TGF-β1), nephrin and Wilms’ tumor-1 (WT-1) as markers to estimate fibrosis development^28^ and podocyte damage^29^,^30^, respectively (Fig. 3). As anticipated, the TGF-β1-positive staining area in the kidneys was far more extensive in *db/db* mice (8.31 ± 0.54%) than in *db/m* mice (1.23 ± 0.22%). CA administration notably reduced the TGF-β1-positive staining area (3.94 ± 0.21%) in *db/db* mice. The expressions of glomerular nephrin and WT-1 were notably decreased in diabetic mice compared with controls. However, treatment with CA significantly ameliorated these changes. Similarly, STZ-diabetic rats demonstrated prominent TGF-β1 expression in the renal cortex (Supplementary Fig. S2). CA treatment caused a dose-dependent reduction of TGF-β1 expression in diabetic rats. The number of podocytes in STZ-diabetic rats was significantly reduced. CA administration notably increased the nephrin and WT-1-positive staining area, but not as markedly as enalapril treatment.

These results showed that CA protects kidneys from damage in type 1 and type 2 models of diabetes.

### Effects of CA on GMC proliferation *in vivo* and *in vitro*

**Figure 4:**
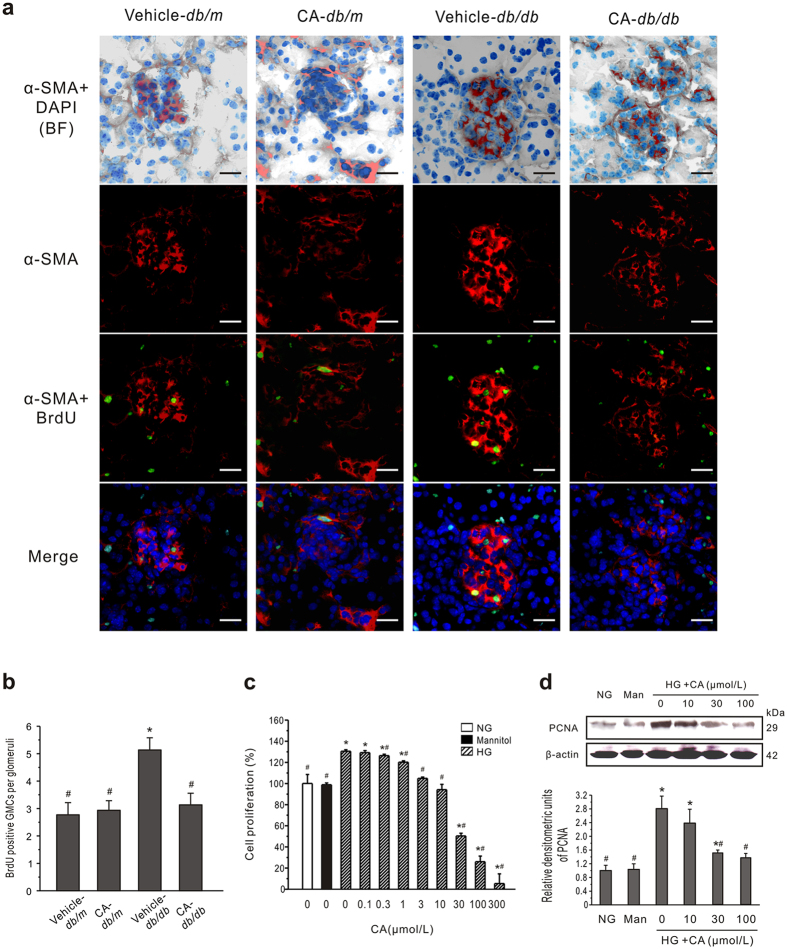
CA inhibits the GMCs proliferation both *in vivo* and *in vitro*. (**a**) Confocal findings in kidney tissue. Nuclear BrdU incorporation shows relative proliferation of GMCs in *db/db* mice. Green, BrdU positive nuclei; red, α-SMA (showing GMCs); blue, DAPI; BF, bright field. Scale bars: 50 μm. (**b**) Quantitation of BrdU positive nuclei in α-SMA-positive segments in each glomeruli. Cells were identified as proliferating mesangial cells if they showed positive nuclear staining for BrdU and if the nucleus was completely surrounded by cytoplasm positive for α-SMA. Data are presented as mean ± SEM (n = 30). *P < 0.05 *vs*. vehicle-treated *db/m* mice; ^#^*P* < 0.05 *vs*. vehicle-treated *db/db* mice. (**c**) Growth-inhibitory effects of CA on GMCs were measured by MTT assay. Mannitol (19.5 mmol/L, Man) was used as an osmotic control. The assay was done with six replicates and repeated three times. (**d**) Western blot analysis of PCNA expression in cultured GMCs treated with NG or HG plus CA for 24 h. Typical results are depicted in upper panel along with statistical analysis of PCNA to β-actin expression in lower panel (n = 3). *P < 0.05 *vs*. NG control; ^#^*P* < 0.05 *vs*. HG control.

Inhibited mesangial expansion was a feature in CA-treated diabetic models. To study the process of proliferation of GMCs *in vivo*, mice were injected with 5-bromo-2′-deoxyuridine (BrdU) and kidneys were harvested for immunostaining. Based on examination of diabetic renal tissue stained with α-smooth muscle actin (α-SMA), there was a marked increase in number and enlargement of α-SMA positive cells in the glomerulus compared to *db*/*m* mice (Fig. 4a). In contrast, sections from the CA-treated diabetic group showed a decrease in α-SMA-positive cell mass and BrdU signal compared to *db*/*db* mice. To assess proliferation more quantitatively, the number of BrdU positive α-SMA nuclei in glomeruli was determined for three animals per group (Fig. 4b). There was a significantly increased number of BrdU-positive GMCs per glomerulus in *db*/*db* mice (5.13 ± 0.45) compared to *db*/*m* mice (2.77 ± 0.45, *P* < 0.05). CA treatment obviously reduced the BrdU-positive number (3.13 ± 0.43) in *db*/*db* mice, whereas it had no significant effect in *db*/*m* mice (2.93 ± 0.35).

To further examine the effect of CA on GMCs *in vitro*, mouse GMCs were cultured and an MTT assay was used to detect cell proliferation. There was obvious proliferation of cells cultured under HG conditions for 48 h (*P* < 0.05) (Fig. 4c). The proliferation rates of the hypertonic and normal glucose groups were not significantly different, indicating that the abnormal cell proliferation induced by HG was not caused by high osmotic pressure. Compared with the HG group, the proliferation rates of cells treated with CA at concentrations of 0.3 to 300 μmol/L were significantly decreased (*P* < 0.05) and the effect was concentration dependent.

**Figure 5:**
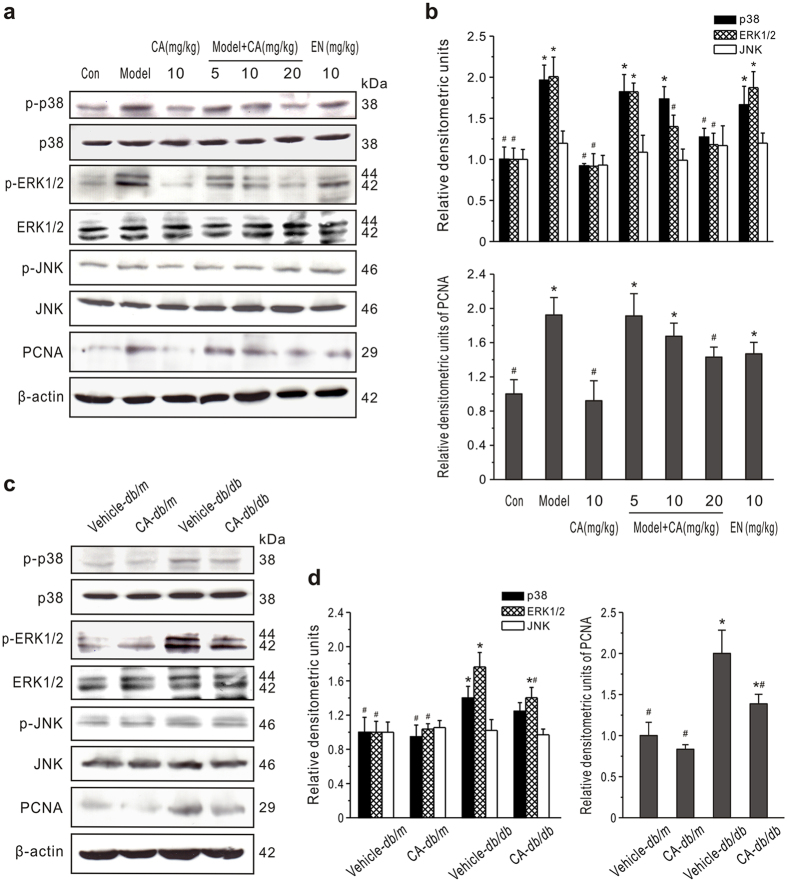
Effect of CA on MAPK expression in renal cortexes. (**a,b**) MAPK and PCNA expression in type 1 diabetic rats. CA and enalapril (EN) were administered daily by gavage to diabetic (model) or normal (Con) rats for 8 weeks. ERK1/2, JNK, p38 and PCNA levels were measured by western blot analysis. Representative western blots are shown (**a**). Densitometric quantification of phosphorylated ERK1/2, p38 or JNK to total ERK1/2, p38 or JNK (*upper panel*) and PCNA to β-actin (*lower panel*) are summarized (**b**). *P < 0.05 *vs*. Con; ^#^*P* < 0.05 *vs*. model. (**c,d**) Effects of CA on MAPK and PCNA expression in *db/db* or *db/m* mice. A typical display is depicted (**c**) along with statistical analysis (**d**) of the changes in the proteins. *P < 0.05 *vs*. vehicle-treated *db/m* mice; ^#^*P* < 0.05 *vs*. vehicle-treated *db/db* mice.

In addition, levels of proliferating cell nuclear antigen (PCNA), highly correlated with cell proliferation^31^, were determined by western blotting. Consistently, concentration-dependent downregulation of PCNA induced by CA was observed in GMCs (Fig. 4d). Likewise, CA significantly inhibited PCNA expression in the renal cortex of STZ-treated rats and *db*/*db* mice (Fig. 5).

### A inhibits ERK and p38 MAPK activation

To investigate whether the MAPK pathway is involved in the CA-induced cell proliferation, we examined expression of ERK1/2, JNK and p38 in renal cortex by western blotting. As shown in Fig. 5, the expression of phosphorylated ERK1/2 and p38 in both type 1 and type 2 DM models increased significantly. Treatment of normal animals with CA did not produce obvious changes in phosphorylated ERK1/2 or p38 expression, while administration of CA to diabetic animals significantly suppressed the phosphorylation of ERK1/2 and p38 (*P* < 0.05). However, there was no significant difference in phosphorylated JNK (p-JNK) expression between diabetic and normal animals, and the treatment with CA did not alter the level of p-JNK in renal cortexes. These data suggested that CA effectively inhibits the ERK1/2 and p38 activation in kidneys in the diabetic state.

**Figure 6:**
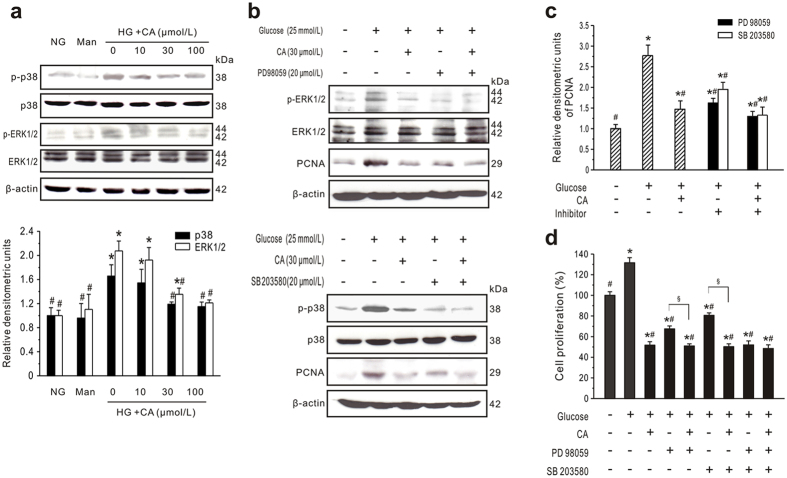
CA inhibits GMCs proliferation *via* ERK1/2 and p38 MAPK activation. (**a**) Western blot analysis of ERK1/2 and p38 expression in cultured GMCs treated with normal glucose (NG) or high glucose (HG) plus CA for 24 h. Mannitol (19.5 mmol/L, Man) was used as an osmotic control. Typical results are depicted in upper panel along with statistical analysis of phosphorylated ERK1/2 or p38 relative to total protein expression in lower panel (*n* = 3). (**b,c**) CA inhibits MAPK activation. GMCs cultured in NG or HG were preincubated with inhibitors of ERK1/2 (PD98059, 20 μmol/L) or p38 MAPK (SB203580, 20 μmol/L) for 30 min followed by treatment with CA (30 μmol/L) for 24 h. Representative western blots (**b**) and statistical analysis of PCNA to β-actin expression (**c**) are shown (*n* = 3). (**d**) CA inhibits GMCs proliferation *via* MAPK activation. GMCs were preincubated with PD98059 and/or SB203580 for 30 min followed by treatment with CA (30 μmol/L) for 48 h. Cell proliferation was measured by MTT assays (*n* = 6). *P < 0.05 *vs*. NG control; ^#^*P* < 0.05 *vs*. HG control; ^§^*P* < 0.05 *vs*. inhibitor without CA.

Next, to investigate the cellular effect of CA, cultured GMCs were incubated with HG plus CA for up to 24 h. HG treatment significantly increased p-p38 and p-ERK1/2 expression (Fig. 6a). In contrast, treatment of GMCs with CA resulted in a concentration-dependent inhibition of p-p38 and p-ERK1/2 expression. These results indicate that the ERK1/2 and p38 pathways might be effectively blocked by CA in HG-treated GMCs.

### CA inhibits GMC proliferation via ERK1/2 and p38 MAPK pathways

To further investigate the signaling cascade between HG-induced cell proliferation and MAPK pathways, inhibitors of the ERK1/2 (PD98059) and p38 (SB203580) signaling molecules were used. After PD98059 or SB203580 treatment, PCNA expression was significantly inhibited compared with the HG group (*P* < 0.05), but CA had a more powerful inhibitory effect on PCNA expression than PD98059 or SB203580 (Fig. 6b,c). Consistent with the PCNA data, PD98059 and SB203580 significantly decreased HG-induced cell proliferation (Fig. 6b,d). Importantly, the proliferation ratio of cells treated with PD98059 or SB203580 plus CA was significantly lower than the ratio of cells treated with PD98059 or SB203580 alone. In addition, compared with cells preincubated with PD98059 plus SB203580 (51.02 ± 3.679%), further CA treatment (48.42 ± 3.70%) did not significantly enhance the inhibition (*P* < 0.05). Taken together, these data suggest that CA inhibits GMC proliferation *via* the ERK1/2 and p38 MAPK pathways.

### CA inhibits ROS generation and expression of isoforms and subunits of NADPH oxidase

**Figure 7:**
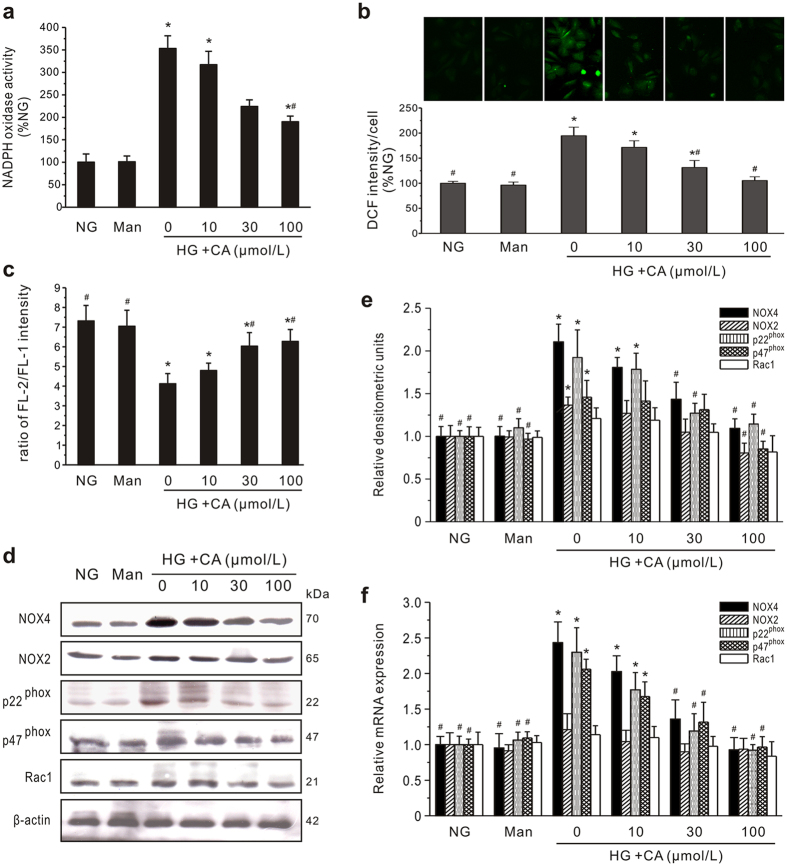
Effects of CA on NADPH oxidase activation, ROS generation, mitochondria membrane potential (Δψm) and expression of isoforms and subunits of NADPH oxidase in GMCs. (**a**) NADPH oxidase activity. Cultured GMCs treated with normal glucose (NG) or high glucose (HG) for 24 h. Mannitol (19.5 mmol/L, Man) was used as an osmotic control. After pretreatment of CA at different concentrations (10, 30, and 100 μmol/L) for 24 h, NADPH oxidase activity was measured. The data were normalized to control cells. (**b**) Intracellular ROS production. GMCs treated with NG or HG plus CA for 24 h. Cells were stained with ROS-sensitive dye DCF-DA for 60 min at 37 °C, then observed under a fluorescence microscope (*upper panel*). All data were normalized to control cells (*lower panel*). (**c**) Effect of CA on Δψm. GMCs were stimulated with HG plus CA for 24 h, and Δψm was measured by JC-1 labeling with flow cytometry (n = 4). (**d–f**) Protein and mRNA expression of NADPH isoforms and NOX-associated subunits. GMCs treated with NG or HG plus CA for 24 h. Expression of proteins including NOX4, NOX2, p22^phox^, p47^phox^ and Rac1 was detected by western blot analysis. Representative western blots (**d**) and statistical analysis (**e**) of each protein’s expression relative to β-actin are showed. Relative amounts of mRNA (**f**) were determined by real-time PCR using 18S as an endogenous control. The experiments were repeated three times. *P < 0.05 *vs*. NG control; ^#^*P* < 0.05 *vs*. HG control.

To investigate the possibility of CA inhibiting oxidative stress, we examined NADPH oxidase activity and ROS generation. Incubation with CA resulted in a concentration-dependent decrease in NADPH oxidase activity (Fig. 7a) and ROS generation (Fig. 7b) compared to the HG group. Using flow cytometry with DHE, DCF-DA and DAF-2/DA, the intracellular superoxide anions (·O^2−^), H_2_O_2_ and NO were further quantified. The HG-induced productions of ·O^2−^ and H_2_O_2_ were inhibited by CA in concentration-dependent manners, respectively (Supplementary Fig. S3). Differently, NO production was decreased in HG media as compared with normal glucose media (*P < *0.05). CA (30 and 100 μmol/L) treatment concentration-dependently increased the levels of NO to the levels similar to those observed in normal glucose media.

As the major sites for ROS production, mitochondria are particularly susceptible to oxidative damage, such as hyperpolarization of the mitochondria membrane potential (Δψm), and their dysfunction promotes excessive ROS production^32^. Thus, we examined the effect of CA on Δψm using flow cytometry. As shown in Fig. 7c, Δψm was significantly degraded by HG stimulation, while treatment with CA concentration-dependently prevented the collapse of Δψm.

Further immunoblotting and qPCR analysis determined the expression of two major isoforms of NADPH oxidase and NOX-associated subunits including p47^phox^, Rac1 and p22^phox^ (Fig. 7d–f). Remarkably, the levels of NOX4 expression were increased to 2.11 ± 0.24 (protein) or 2.53 ± 0.29 (mRNA) relative to the normalized quiescent levels (protein, 1.00 ± 0.12; mRNA, 1.00 ± 0.11) after HG treatment, which was the highest level for these NADPH oxidase isoforms and NOX-associated subunits. In addition, although HG increased NOX2 and Rac1 expression, there was no significant difference between the HG group and the control group. Treatment with CA led to the concentration-dependent inhibition of NOX4, NOX2, p22^phox^ and p47^phox^ expression at both the transcriptional and translational levels. NOX4, NOX2, p22^phox^ and p47^phox^ protein expression in cells treated with 100 μmol/L CA were decreased to 1.09 ± 0.11, 0.80 ± 0.12, 1.15 ± 0.11 and 0.85 ± 0.09, respectively, values significantly lower than for HG control cells (2.11 ± 0.24, 1.31 ± 0.15, 1.92 ± 0.32 and 1.46 ± 0.14, *P* < 0.05).

### CA-inhibited GMC proliferation mediated by NADPH/ERK1/2 and p38 MAPK signaling pathways

**Figure 8:**
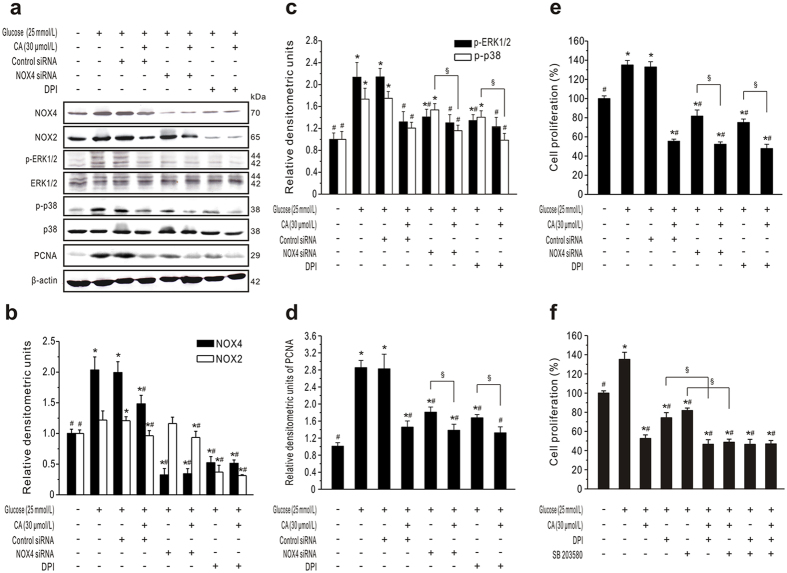
CA inhibits GMC proliferation mediated by NADPH/ERK1/2 and p38 MAPK signaling pathways. (**a–e**) CA inhibits GMC proliferation *via* NADPH. GMCs serum-starved for 24 h were transiently transfected with NOX4 or control siRNA; 48 h later, the cells were pretreated with NADPH oxidase inhibitors DPI (10 μmol/L) for 30 min, followed by incubation with normal glucose (NG) or high glucose (HG) in the presence or absence of CA (30 μmol/L). To assess the inhibitory effect of CA on protein expression or cell proliferation, GMCs were further incubated for 24 h or 48 h, respectively. Representative western blots and statistical analysis are shown in (**a–d**). (**e**) Cell proliferation was measured by MTT assays. (**f**) CA inhibits GMC proliferation co-regulated by NADPH and p38 MAPK. GMCs were pretreated with DPI (10 μmol/L) and/or SB203580 (20 μmol/L) for 30 min, followed by treatment with NG or HG in the presence or absence of CA (30 μmol/L) for 48 h. Cell proliferation was measured by MTT assays (*n* = 6). *P < 0.05 *vs*. NG control; ^#^*P* < 0.05 *vs*. HG control; ^§^*P* < 0.05 *vs*. inhibitor/siRNA without CA.

We therefore employed NOX4 siRNA and an NADPH oxidase inhibitor, DPI, to inhibit NOX4 expression and block the activation of NADPH signaling respectively (Fig. 8a,b). DPI treatment revealed that both NOX4 and NOX2 expression were greatly suppressed in HG-stimulated GMCs. PCNA expression and proliferation in GMCs treated with HG were reduced by NOX4 siRNA and DPI respectively (Fig. 8d,e). Interestingly, there was a significant difference in PCNA expression and proliferation between NOX4 siRNA and DPI groups (*P* < 0.05), which suggests that NOX4 is the major isoform involved in the GMC proliferation, and NOX2 or other NOX-associated subunits such as p47^phox^ and p22^phox^ also participated in regulation. Moreover, when cell were treated with CA, further suppressing NOX4 or NADPH activation did not significantly enhance the decrease in PCNA expression and cell proliferation. These data suggested that NADPH oxidase is necessary for the effect of CA on proliferation of GMCs.

We also demonstrated the role of NADPH on the MAPK pathway in CA-treated GMCs. As shown in Fig. 8a,c, inhibition of NOX4 or NADPH significantly decreased the phosphorylation of ERK1/2 and p38 MAPK induced by HG (*P* < 0.05). This effect was more pronounced in DPI-treated cells than NOX4 siRNA-treated cells. These data demonstrated that the NADPH pathway is upstream of ERK1/2 and p38 MAPK in GMCs under hyperglycemic conditions. More importantly, there was no significant difference in the level of p-ERK1/2 in DPI-treated cells compared to CA plus DPI co-treated GMCs. But the expression of p-p38 MAPK in CA plus NOX4 siRNA or DPI-treated cells was markedly lower than in NOX4 siRNA or DPI-treated cells alone, which suggests that CA regulate p38 MAPK activation at least partly independently of the NADPH pathway.

Moreover, Fig. 8d,e show that CA can further downregulate PCNA expression and inhibit cell proliferation compared to NOX4 siRNA or DPI-treated cells alone (*P* < 0.05). These results indicated that CA-inhibited cell proliferation is mediated partly by the NADPH pathway. To determine whether p38 MAPK and NADPH are co-regulated by CA to inhibit cell proliferation, GMCs were pretreated with inhibitors SB203580 and/or DPI in the presence or absence of CA. The inhibition by SB203580 plus DPI (Fig. 8f) was similar to that of CA. Thus, CA inhibits NADPH oxidase and phosphorylation of p38 MAPK, resulting in the inhibition of GMC proliferation.

## Discussion

Clinically, DN is characterized by hyperglycemia, hypertension, progressive albuminuria, decline in glomerular filtration rate, and a high risk of cardiovascular morbidity and mortality. Presently, treatment of patients with DN is limited to angiotensin converting enzyme inhibitors and angiotensin receptor blockers, but renal protection by these drugs is suboptimal^3^. To identify novel anti-DN agents, recent investigation has focused on targets upregulated by hyperglycemia or other signal pathways promoting the progression of DN, such as oxidative stress, inflammation or the endothelin system.

In the present study, the renoprotective effects of CA were assessed using STZ-treated rats and *db/db* mice. We found that CA markedly ameliorated the increase in the levels of urinary albuminuria, Cre and BUN. Clinically, these renal functional parameters are highly correlated with structural changes, especially with the degree of glomerular mesangial expansion in both type 1 and type 2 diabetes. Mesangial expansion is the lesion leading to the loss of glomerular filtration rate, and is present in DN patients before the onset of clinical manifestations^33^,^34^. Amelioration of mesangial expansion is now considered one of the targets in the prevention and retardation of DN^35^. Based on histopathological analysis, we confirmed that CA significantly inhibits glomerular hypertrophy and mesangial expansion. These data suggest that therapy with CA may be useful in preventing the renal damage of DM. What, then, is the mechanism by which CA protects the kidney against mesangial expansion?

It has been demonstrated that mesangial hypercellularity precedes an increase in the level of extracellular matrix proteins and glomerular sclerosis^36^. The level of intracellular glucose is strongly associated with GMC proliferation, so GMCs cultured in HG are employed to study DN at the cellular level^37^. In the present study, we observed that CA markedly inhibits GMC proliferation in *db*/*db* mice. A further *in vitro* study demonstrated that CA inhibits HG-induced GMC proliferation in a concentration-dependent manner, with a half maximal inhibitory concentration (IC50) of 22.50 ± 3.41 μmol/L. We thus selected a dose of 30 μmol/L for subsequent experiments to determine the signaling pathways in GMCs.

HG induces ROS, which play a key role in GMC hyperplasia. A gp91^phox^ homologue, NOX4, is highly expressed in human^38^ and rat kidneys^39^ in DN. Similar increases were detected in GMCs exposed to HG, which is associated with an increase in cellular and mitochondrial ROS production^40^. Recent studies have shown that inhibition of NOX4 oxidase expression with antisense oligonucleotides or by genetic deletion markedly attenuates diabetes-induced oxidative stress and mesangial matrix expansion in glomeruli of diabetic animals^41^. Due to the important contribution of NOX4 to renal damage, we measured NOX4 expression in the kidney cortex. We observed a significant downregulation of NOX4 expression in CA-treated diabetic groups (Supplementary Fig. S4), suggesting that CA may disrupt NADPH oxidase activity.

NADPH oxidase in the resting state becomes activated to produce superoxide upon interaction with cytoplasmic components such as p22^phox^, p47^phox^, p67^phox^ and Rac1 (a small GTPase). p22^phox^ could interact with NOX2 and/or NOX4. The isoforms p47^phox^, p67^phox^, and Rac1 are associated with NOX2 activity^42^. Previous study confirmed that HG elicits ROS generation mainly *via* NOX4-based NADPH oxidase and the p22^phox^ subunit in GMCs^43^. However, the other NOX and regulatory subunits that are implicated in the development of DN and growth of GMCs remain to be identified. Some studies detected NOX2 and its regulatory subunit p47^phox^ in diabetic glomeruli^44^. Importantly, inhibition of p47^phox^ blunts HG-induced extracellular matrix accumulation^45^. p47^phox^ genetic deletion in type 1 diabetic mice attenuated glomerular injury^46^. These data indicate that p47^phox^ is also a determinant for the promotion of DN. In this study, expression of NOX4, p22^phox^ and p47^phox^, but not NOX2 or Rac1, was significantly increased in HG-stimulated GMCs. We subsequently found that CA ameliorated their expression in a concentration-dependent manner. These results suggest that CA might have an inhibitory effect on HG-induced GMC prolifiration *via* NOX4, p22^phox^ and p47^phox^.

Mounting evidence suggests that NADPH regulates the MAPK signaling pathway to mediate cell proliferation. In this study, we also used specific inhibitors to validate upstream or downstream signaling cascades involved in CA-inhibited cell proliferation. The phosphorylation levels of ERK1/2 and p38 MAPK were remarkably inhibited by treatment with DPI. Interestingly, DPI suppressed HG-induced ERK 1/2 phosphorylation similarly to CA, while CA inhibited p38 MAPK phosphorylation more than DPI did. On the other hand, using the p38 MAPK inhibitor SB203580 plus DPI could inhibit cell proliferation to a similar extent to CA. These data indicate that NADPH regulates the ERK1/2 signaling pathway to mediate cell proliferation. Moreover, it seems that CA suppresses HG-induced cell proliferation by inhibiting NADPH and p38 MAPK simultaneously.

Hyperglycemia is a well-known factor influencing its progression. Intensive glycemic control to prevent the progression of DN is recommended (American Diabetes Association, 2013). Previous studies showed that CA has a lowering effect on postchallenge plasma glucose levels in humans *in vivo*^9^, through enhancing glucose uptake, facilitating glucose transporter isoform 4 translocation and inhibiting the enzymatic activities of several diabetes-related non-receptor protein tyrosine phosphatases, such as PTP1B, T-cell-PTP, src homology phosphatase-1 and src homology phosphatase-2^47^. In this study, the blood glucose lowering effects were observed at 4, 6 and 8 weeks of treatment with CA, suggesting that stable long-term glycemic control can be achieved by CA treatment. Collectively, the therapeutic benefit of CA on DN is multifactorial, not only by inhibiting HG-induced GMC proliferation via NADPH and p38 MAPK signaling but also by improving blood glucose levels in diabetic animals.

The present study demonstrates that CA protects renal damage in type 1 and 2 diabetic animals. CA inhibits the proliferation of diabetic glomerular mesangial cells via NADPH/ERK1/2 and p38MAPK signaling pathways. These findings suggest that CA can be used therapeutically to prevent the onset and progression of diabetic nephropathy.

## Methods

### Diabetic models

Two animal models of diabetes were used in this study. Animals were provided a temperature- and humidity-controlled SPF environment with a 12 h light-dark cycle at the Center of Experimental Animals of the Fourth Military Medical University (FMMU, Xi’an, China) and, maintained on standard chow and water ad libitum in sterile standard cages with bedding material.

The first was STZ-induced diabetic rats. Male Sprague-Dawley (SD) rats (180 to 200 g) were purchased from the Center of Experimental Animals of FMMU and were housed to acclimatize for one week. The blood glucose levels and body weights of all animals were measured at the beginning of the study. The rats were fasted overnight, and then injected intraperitoneally once with STZ at a dose of 60 mg/kg diluted in citrate buffer (0.1 mol/L, pH 4.5) to induce diabetes. Two days later, rats with blood glucose over 16.7 mmol/L were confirmed to be in diabetic state. The rats were randomly divided into the following seven groups (*n* = 8 per group) using a table of random numbers, a systematic, physical approach: normal rats (control), diabetic model group, CA-treated group (10 mg/kg/day), CA-treated diabetic groups (5, 10 or 20 mg/kg/day) and enalapril-treated group (10 mg/kg/day). CA and enalapril were dissolved in 0.5% carboxymethylcellulose sodium salt (CMC), and administered daily by gavage to diabetic or CA-control rats for 8 weeks. The rats in both control and model groups were given the same volume of vehicle (0.5% CMC) by gavage.

The second animal model was based on diabetic *db*/*db* mice. Six-week-old male diabetic C57BLKS/JNju *db*/*db* mice (28 to 31 g) and their age-matched non-diabetic *db/m* mice, purchased from Nanjing Biomedical Research Institute (Nanjing, China), were allowed to acclimatize for two weeks before beginning the experiments. Then *db*/*db* and *db/m* mice were randomly divided into the following four groups (*n* = 6 per group) using a table of random numbers, a systematic, physical approach: vehicle-treated *db/m* mice, CA-treated *db/m* mice, vehicle-treated *db/db* mice, and CA-treated *db/db* mice. CA dissolved in 0.5% CMC was administered, 10 mg/kg/day, via gavage to CA-treated mice for 8 weeks.

At the termination of the experiment and after overnight fast, animals were killed by whole blood collection from the abdominal aorta under an aesthesia with pentobarbital sodium. The kidney samples were rapidly excised, weighed and cleaned in ice-cold PBS. One kidney from each animal was fixed in 10% formaldehyde, while the renal cortex was isolated immediately from another one, snap-frozen in liquid nitrogen and stored at −80 °C for western blot and PCR analysis. In this study, the efforts were made to minimize suffering and to reduce the number of animals used. All of the animal care and handling procedures were approved by the Institutional Animal Care and Use Committee of FMMU and all animal experiments were performed in accordance with the approved guidelines.

### Measurement of body weight, kidney index and blood glucose

Body weight of animals was measured at 1-week intervals. The kidney index was calculated according to the formula: kidney index (mg/g) = kidney weight (mg)/body weight (g). Blood was sampled from the tail vein at 1-week intervals, and fasting blood glucose level was measured using a blood glucose meter (IGM-0005A, Infopia Co., Korea).

### Serum and urine measurements

At the last week of medication, urine was gathered for 24 h using metabolism cages. Urinary albuminuria was determined with a commercially available ELISA kit (Exocell, Philadelphia, PA, USA). Cre and BUN were measured using assay kits (Nanjing Jiancheng Bioengineering Institute, Nanjing, China) with a biochemistry analyzer (RT-9600, Rayto, USA).

### Histological examination of the kidney

Renal sections were stained with H&E, PAS and Masson’s trichrome, then observed under an Eclipse 80i microscope (Nikon, Japan). Six glomeruli were randomly selected from five sections in each group, and were analyzed using Image-Pro Plus 6.0 software (Media Cybernetics, Silver Spring, MD, USA). The glomerular volumes were calculated using the formula: glomerular volume = Area^1.5^ × 1.38/1.01^48^. The MEI was scored at four levels as described previously^49^, with the index scores defined as follows: 0, normal glomeruli; 1, matrix expansion occurring in up to 50% of glomeruli; 2, matrix expansion occurring in 50–75%; 3, matrix expansion occurring in 75–100%. The level of glomerular fibrosis was quantitatively measured with Masson’s trichrome staining of 30 glomeruli. The fibrosis region was measured by extracting the area of blue component (fibrosis was stained as blue within the Tri-chrome staining) using Image-Pro Plus 6.0 software. The fibrosis level was expressed as the ratio between stained blue fibrotic tissue and total measured area.

### Immunohistochemical analysis

Immunohistochemical analysis was performed on 4-mm-thick paraffin-embedded kidney sections. After dewaxing and rehydrating, endogenous peroxidase was quenched for 10 min with 0.3% H_2_O_2_ in methanol. Heat-mediated antigen retrieval and enzymatic techniques were performed according to recommendations for the specific antibodies. A blocking step was performed using 10% normal goat serum. The kidney sections were incubated overnight at 4 °C with anti-collagen IV (Abcam, Cambridge, MA, USA), nephrin (Santa Cruz, CA, USA), TGF-β1 (Santa Cruz) or WT-1 (Abcam) primary antibodies. After washing with TBS and incubation with biotinylated secondary antibodies and Vectastain ABC reagent (Vector Laboratories Inc., Burlingame, CA, USA), the samples were visualized using diaminobenzidine staining and counterstaining with hematoxylin. Negative controls had the primary antibodies omitted. Sections were examined using a Nikon Eclipse 80i microscope (magnification, ×400). Immunostaining signals were highlighted and quantified using Image-Pro Plus 6.0 software. Areas with positive staining were quantified and expressed as a percentage of the entire area (n = 20–30).

### 5-bromo-2′-deoxyuridine (BrdU) detection

Proliferation in regions of the kidney was assayed using BrdU incorporation as previously described^50^. Briefly, BrdU (100 mg/kg) was injected intraperitoneally into mice 4 h before killing. Kidneys were frozen in OCT embedding compound and 10-μm sections were cut on a Leica cryostat. Following acetone fixation and permeabilizing with 0.1% Triton X-100, the sections were immunostained with anti-BrdU (Abcam) and α-SMA (Abcam) antibody after blocking with 10% goat serum. After further washing, the sections were incubated with FITC-conjugated (Jackson Immuno-Research Laboratories, West Grove, PA, USA) and Cy3-conjugated (Jackson) secondary antibodies. DAPI solution was used to counterstain the nucleus. Immunofluorescence images were obtained using a confocal microscope (FV1000, Olympus, Japan).

### Cell Culture

GMCs were separated from the glomeruli of C57BL/6 as previously described^51^. They were cultured at 37 °C in a humidified 5% CO_2_ incubator in DMEM, 5 mmol/L D-glucose, supplemented with 10% fetal bovine serum (FBS), 100 U/mL penicillin, 100 μg/mL streptomycin. In order to avoid contamination by residual epithelial or endothelial cells, experiments were performed between the 5th and 10th passage. GMCs showed normal morphology and function in these passages. Confluent cells were rendered quiescent by serum starvation in DMEM with 0.5% FBS for 24 h before treatment with chemicals or stimulation as specified in figure legends. For normal glucose (5.5 mmol/L) control treatment, 19.5 mmol/L mannitol was added to the medium as an osmotic control. For HG treatment, the concentration of D-glucose was 25 mmol/L.

### Cell proliferation assay

Cell proliferation was determined using the MTT assays^52^. Exponentially growing cells (3 × 10^3^/well) were planted in 96-well plates, serum starved for 24 h and incubated with normal glucose (NG, 5.5 mmol/L) or high glucose (HG, 25 mmol/L) plus CA (0–300 μmol/L) for 48 h. As an osmotic control, 19.5 mmol/L mannitol (MAN) was added to the NG medium. The proliferation rate was calculated using the following formula: proliferation rate = OD (experimental group) − OD (control group)/OD (control group).

### Transient transfection

Small interfering (si)RNA against mouse NOX4 (Santa Cruz Biotechnology, Santa Cruz, CA, USA) or control siRNA was transfected into GMCs using Lipofectamine Plus reagent (Invitrogen-BRL, Carlsbad, CA, USA) following the protocols provided by the manufacturer. After 48 h of transfection, the cells were treated with the chemicals specified in figure legends and harvested for western blotting.

### Western blot analysis

NOX4 (Abcam, Cambridge, MA, USA), NOX2 (Abcam), p22^phox^ (Santa Cruz Biotechnology), p47^phox^ (Santa Cruz Biotechnology), Rac1 (Abcam), phospho-ERK1/2 (Cell Signaling Technology (CST), Danvers, MA, USA), ERK1/2 (CST), phospho-p38 (CST), p38 (CST), phospho-JNK (CST), JNK (CST), PCNA (Abcam) and β-actin (Sigma) protein levels were determined by western blot analysis as described previously^53^.

### Measurement of ROS and intracellular ·O^2−^, H_2_O_2_ and NO

Generation of intracellular ROS was measured with the fluoroprobe 2′,7′-dichlorofluorescein diacetate (DCF-DA) as previously described^54^. Briefly, GMCs were treated with various concentrations of CA (0–100 μmol/L) for 24 h, and then incubated for 60 min with 10 μmol/L DCF-DA. Fluorescence intensity was measured by a fluorescence microscope (Nikon). Average intensity for each group of cells was determined using Image-Pro Plus 6.0 software, and values were expressed as relative to control. An average of 10 cells/field and 4 random fields were chosen for each group. Furthermore, intracellular ·O^2−^, H_2_O_2_ and NO were measured using flow cytometry as described previously^55^. Briefly, GMCs grown in medium containing 5 or 25 mM glucose were treated with various concentrations of CA (0–100 μmol/L) for 24 h. Dihydroethidium (DHE) and 2′,7′-dichlorofluorescein diacetate (DCF-DA) were used to detect intracellular ·O^2−^ and H_2_O_2_, respectively. DHE (160 mM) or DCF-DA (20 mM) was added to cell suspensions (10^6^ cells) which were then incubated at 37 °C for 30 min in the dark. The NO-sensitive fluorescent probe 4,5-diaminofluorescein-2 diacetate (DAF-2/DA, 2 μM) was added to the cell suspension, and the cells were incubated at 37 °C for 180 min in the dark. Cells were then washed, resuspended in PBS, and maintained on ice for immediate detection with a flow cytometer (FACSCalibur, BD Biosciences, Franklin Lakes, New Jersey, USA). The excitation wavelength was 488 nm. DHE, DCF and DAF fluorescence were detected using 585/42 nm (DHE) and 530/30 nm (DCF and DAF) bandpass filters. Data were analyzed using CellQuest software (BD Biosciences), and expressed as the geometric mean fluorescence intensity (MFI, n = 4).

### Assessment of Δψm

Δψm was monitored with the dye, 5,5′,6,6′-tetrachloro-1,1′,3,3′-tetraethyl- benzimidazolylcarbocyanine iodide (JC-1, Molecular Probes) using flow cytometric analyses as described previously^56^. GMCs cultured in medium containing 5 or 25 mM glucose were treated with various concentrations of CA (0–100 μmol/L) for 24 h. Cells were harvested by trypsinization, loaded with JC-1 dye (1 mg/L) at 37 °C for 30 min in the dark, washed in PBS and analyzed by flow cytometry (BD Biosciences). The excitation wavelength was 488 nm, and the emission fluorescence for JC-1 was monitored at 530 nm (FL-1, green fluorescence) and 585 nm (FL-2, red fluorescence). Data were analyzed using CellQuest software and the ratio of FL-2/FL-1 intensity values were used to assess Δψm (n = 4).

### NADPH oxidase assay

NADPH oxidase activity was measured by a lucigenin-enhanced chemiluminescence method as previously described^43^. Briefly, GMCs were treated with CA, washed with PBS, and then homogenized in lysis buffer. A total of 100 μL supernatant of the homogenate was added to 900 μL of 50 mmol/L phosphate buffer (containing 5 μmol/L lucigenin as an electron acceptor, and 100 μmol/L NADPH as an electron donor). Photon emission expressed as relative light units was measured every 30 s for 5 min in a luminometer. Protein concentration was determined using the Bradford assay. A buffer blank was subtracted from each reading. Superoxide production was expressed as the rate of relative chemiluminescence units per minute per microgram of protein, and expressed as fold increase compared to untreated cells. The experiments were repeated three times.

### Real-time PCR

Total RNA was extracted from GMCs using an RNeasy Mini Kit (Qiagen, Valencia, California, CA, USA) according to the manufacturer’s instructions. cDNA was synthesized from 1 μg of total RNA using an iScript cDNA Synthesis Kit (Bio-Rad) and PCR was performed in a 20 μL reaction mixture using SYBR Green PCR Master Mix (Bio-Rad) and analyzed on an iCycler iQ real-Time PCR Detection System (Bio-Rad)^40^. NOX2, NOX4, p22^phox^, p47^phox^, Rac1 and 18S (an endogenous control) primer sequences are shown in the Supplementary Table. The relative amounts of mRNA were determined by ΔΔ*Ct* calculations. Each sample was tested in triplicate.

### Reagents

DMEM and FBS were obtained from Gibco-BRL (Carlsbad, CA, USA). CA (purity > 98%) was purchased from Baoji Chenguang Technology Co. Ltd (Baoji, Shaanxi, China). NADPH was purchased from Roche Diagnostics (Mannheim, Germany). D-glucose, BrdU, STZ, DCF-DA, PD98059, SB203580, diphenylene iodonium (DPI) and other biochemicals were purchased from Sigma-Aldrich (St. Louis, MO, USA).

### Statistical analysis

All reported values were presented as mean ± SEM with *n* denoting the sample size in each group. An unpaired Student’s *t*-test was used to compare data between two groups, whereas one-way ANOVA with Tukey’s post-hoc tests was used for multiple comparisons. Differences were considered significant if the *P*-value was less than 0.05. To minimize the bias, the operators and data analysts were blinded.

## Additional Information

**How to cite this article**: Li, X.-Q. *et al.* Corosolic acid inhibits the proliferation of glomerular mesangial cells and protects against diabetic renal damage. *Sci. Rep.* **6**, 26854; doi: 10.1038/srep26854 (2016).

## Supplementary Material

Supplementary Information

## Figures and Tables

**Table 1:** The effects of CA on fasting blood glucose in STZ-treated rats after treatment (n = 8).

Groups	Blood glucose (mmol/L)				
	0 week	2 week	4 week	6 week	8 week
Control	5.83 ± 0.56^#^	6.07 ± 0.71^#^	6.33 ± 0.66^#^	6.25 ± 0.83^#^	6.48 ± 0.38^#^
Model	17.68 ± 1.59*	19.03 ± 2.01*	19.99 ± 2.14*	21.87 ± 2.41*	26.16 ± 1.42*
Model + CA (5 mg/kg)	17.14 ± 1.98*	18.46 ± 1.91*	18.85 ± 1.97*	19.02 ± 2.29*	21.23 ± 1.76*^,#^
Model + CA (10 mg/kg)	17.95 ± 1.83*	16.53 ± 2.08*^,#^	16.03 ± 1.86*^,#^	15.09 ± 1.69*^,#^	15.81 ± 2.15*^,#^
Model + CA (20 mg/kg)	17.46 ± 1.99*	16.17 ± 1.90*^,#^	15.27 ± 2.25*^,#^	14.32 ± 2.01*^,#^	13.32 ± 1.84*^,#^
Model + Enalapril (10 mg/kg)	18.02 ± 2.03*	18.24 ± 1.88*	18.36 ± 2.13*	20.05 ± 2.24*	24.62 ± 1.52*
CA (10 mg/kg)	5.96 ± 0.64^#^	5.79 ± 0.75^#^	6.19 ± 0.77^#^	6.04 ± 0.51^#^	6.18 ± 0.48^#^

^*^P < 0.05 vs. control; ^#^*P* < 0.05 vs. model.

**Table 2:** The effects of CA on the characteristics and biochemical parameters of STZ-treated rats (n = 8).

Groups	Body weights (g)	Kidney weight (g)	Kidney index (×10^−3^)	Albuminuria (μg/day)	BUN (mmol/L)	Serum creatinine (μmol/L)
Control	486.16 ± 17.43^#^	3.37 ± 0.11^#^	6.95 ± 0.16^#^	14.27 ± 0.87^#^	5.40 ± 0.51^#^	28.57 ± 1.85^#^
Model	353.60 ± 10.77*	3.77 ± 0.19*	10.66 ± 0.35*	94.78 ± 4.52*	12.17 ± 1.35*	45.41 ± 3.50*
Model + CA (5 mg/kg)	387.37 ± 12.91*^,#^	3.68 ± 0.16	9.55 ± 0.47*^,#^	85.98 ± 4.58*	11.50 ± 0.71*	41.10 ± 2.47*
Model + CA (10 mg/kg)	425.20 ± 18.25*^,#^	3.46 ± 0.17	8.18 ± 0.21*^,#^	82.00 ± 4.13*^,#^	11.16 ± 1.11*	38.29 ± 2.20*
Model + CA (20 mg/kg)	446.55 ± 15.98^#^	3.40 ± 0.17	7.65 ± 0.45^#^	72.14 ± 5.21*^,#^	9.14 ± 0.64*^,#^	32.68 ± 3.69^#^
Model + Enalapril (10 mg/kg)	388.97 ± 17.18*^,#^	3.60 ± 0.21	9.25 ± 0.33*^,#^	78.79 ± 3.23*^,#^	11.00 ± 0.82*	38.64 ± 2.60*
CA (10 mg/kg)	480.82 ± 24.19^#^	3.31 ± 0.17^#^	6.87 ± 0.11^#^	15.05 ± 1.59*^,#^	5.52 ± 0.77^#^	28.98 ± 1.87^#^

^*^P < 0.05 vs. control; ^#^*P* < 0.05 vs. model.
