# Supplementary material for: Corosolic acid inhibits the proliferation of glomerular mesangial cells and protects against diabetic renal damage
Source: Sci Rep. 2016 May 27;6:26854. doi: 10.1038/srep26854 (PMC4882506; doi:10.1038/srep26854)
Supplement: Supplementary Information [file srep26854-s1.doc]

Supplementary Information

**Corosolic acid inhibits the** **proliferation of** **glomerular mesangial cells and protects against diabetic renal damage**

Xiao-Qiang Li^1^, Wen Tian^1,2^,Xiao-Xiao Liu^1^, Kai Zhang^1^, Jun-Cheng Huo^2,3^, Wen-Juan Liu^3^, Ping Li^3^, Xiong Xiao^1^, Ming-Gao Zhao^1^, Wei Cao^3^*

^1^*Department of Pharmacology, School of Pharmacy, Fourth Military Medical University, Xi’an 710032, China*

^2^*Cadet Brigade, Fourth Military Medical University, Xi’an 710032, China*

^3^*Department of Natural Medicine & Institute of Materia Medica, School of Pharmacy, Fourth Military Medical University, Xi’an 710032,China*

*Corresponding author: Wei Cao

Department of Natural Medicine & Institute of Materia Medica, School of Pharmacy, Fourth Military Medical University, 169 Changle West road, Xi’an, Shaanxi, 710032,China

Tel.: +86 29 84773752; fax: +86 29 83224790.

E-mail address: [caowei@fmmu.edu.cn](mailto:caowei@fmmu.edu.cn)

Supplementary Table

**Supplementary Table** Primer sequences for real-time PCR.

|  | Forward | Reverse |
| --- | --- | --- |
| *NOX2* | 5’-TGATGTTAGTGGGAGCCGGGATTG-3’ | 5’-TCTGCAAACCACTCAAAGGCATG-3’ |
| *NOX4* | 5’-GGATCACAGAAGGTCCCTAGCAG-3’ | 5’-GCGGCTACATGCACACCTGAGAA-3’ |
| *p22^phox^* | 5′-GGCACCATCAAGCAACCACC-3′ | 5′-CTCATCTGTCACTGGCATTGGG-3′ |
| *p47^phox^* | 5′-CGAAGAAGCCTGAGACATACC-3′ | 5′-ATATCCCCTTTCCTCACCACC-3′ |
| *Rac1* | 5′-CTGAAGTGCGACACCACTGT-3′ | 5′-CAGCAGGCATTTTCTCTTCC-3′ |
| *18S* | 5’-GGCTACCACATCCAAGGAA-3’ | 5’-GCTGGAATTACCGCGGCT-3’ |

Supplementary Figures and Figure Legends


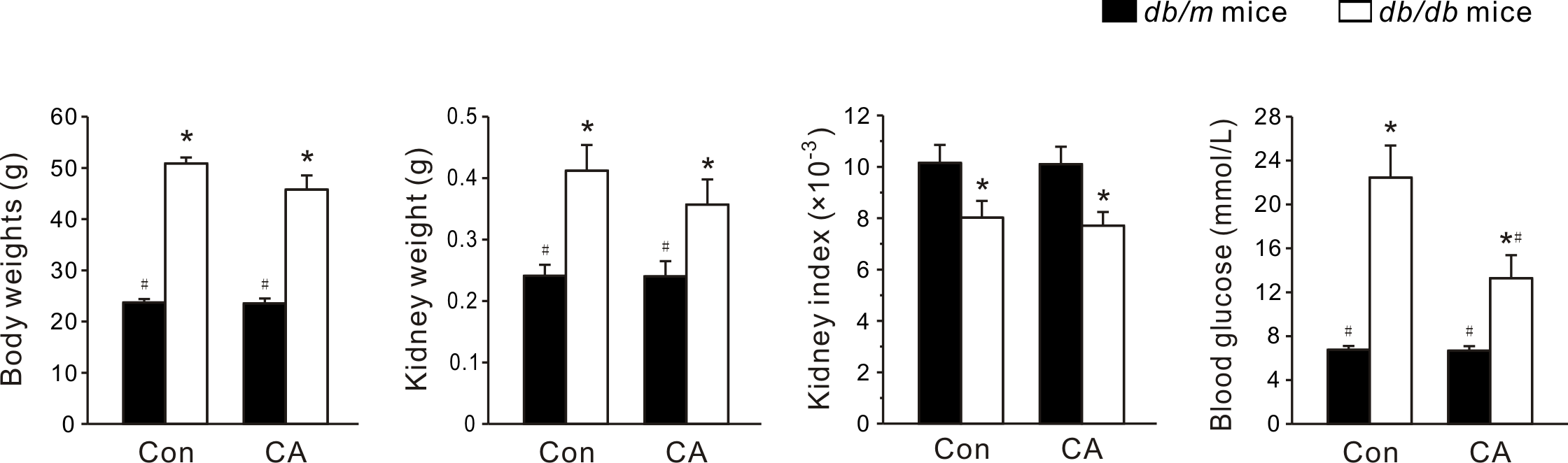


**Supplementary Fig. S1.** Effects of corosolic acid (CA) on the characteristics and biochemical parameters of *db/db* mice. Eight-week-old *db/db* and age-matched *db/m* mice (control, Con) were treated with CA (10 mg/kg/day) or vehicle (0.5% CMC) by gavage for 8 weeks. Body weights, kidney weights, kidney index (mg/g) and blood glucose were measured. Data are presented as mean±SEM **P*<0.05 *vs*. vehicle-treated *db/m* mice; ^#^*P*<0.05 *vs*. vehicle-treated *db/db* mice.


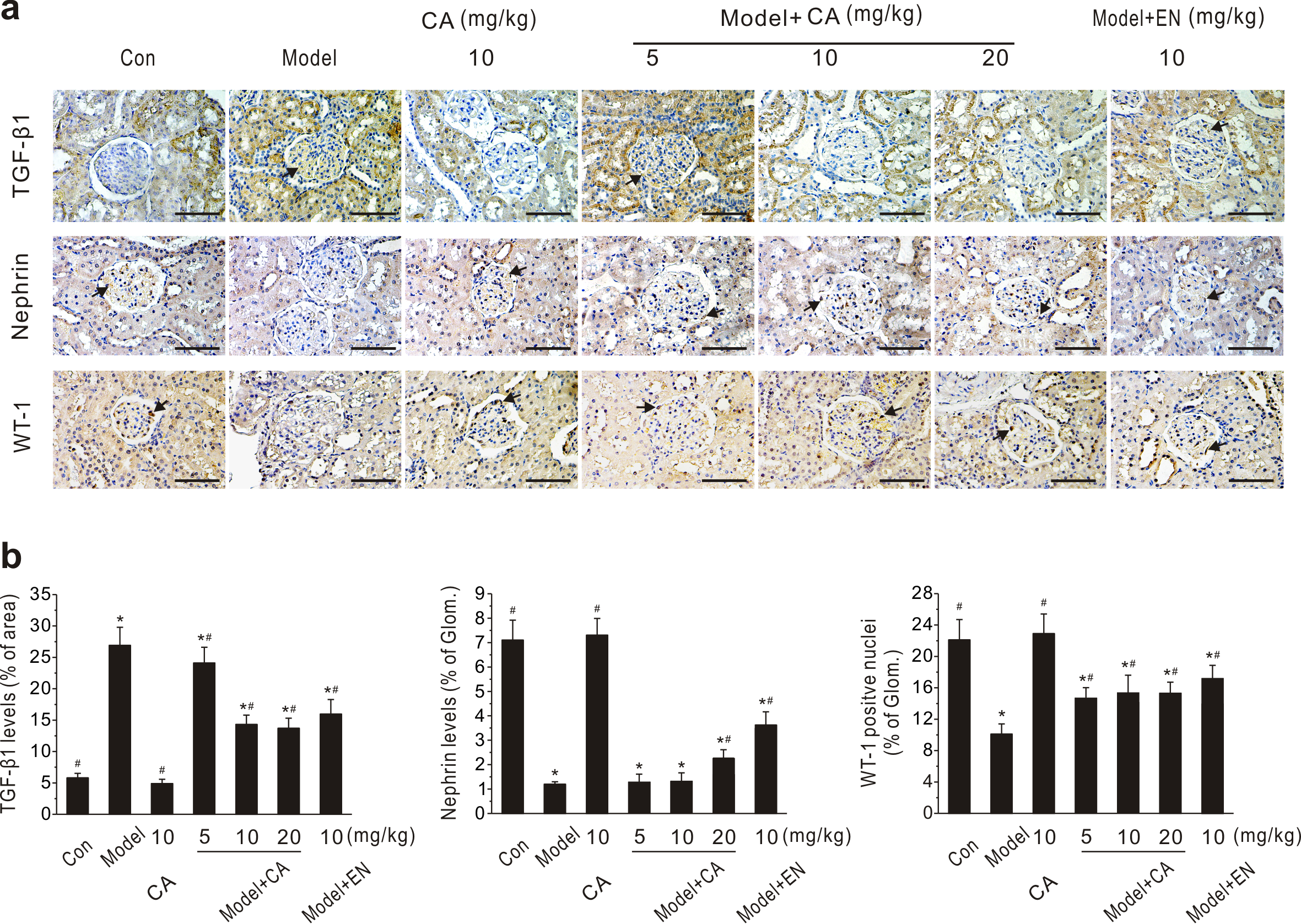


**Supplementary Fig. S2.** Effects of CA on glomerular TGF-β1, nephrin and Wilms’ tumor-1 (WT-1) levels in diabetic rats. (a) Representative immunohistochemical images. Scale bars: 100 μm. (b) Quantitative assessments of immunohistochemical staining in the kidneys. Twenty glomeruli were randomly selected from four kidneys in each group. Data are presented as mean ± S.E.. **P*<0.05 *vs*. control; ^#^*P*<0.05 *vs*. model.


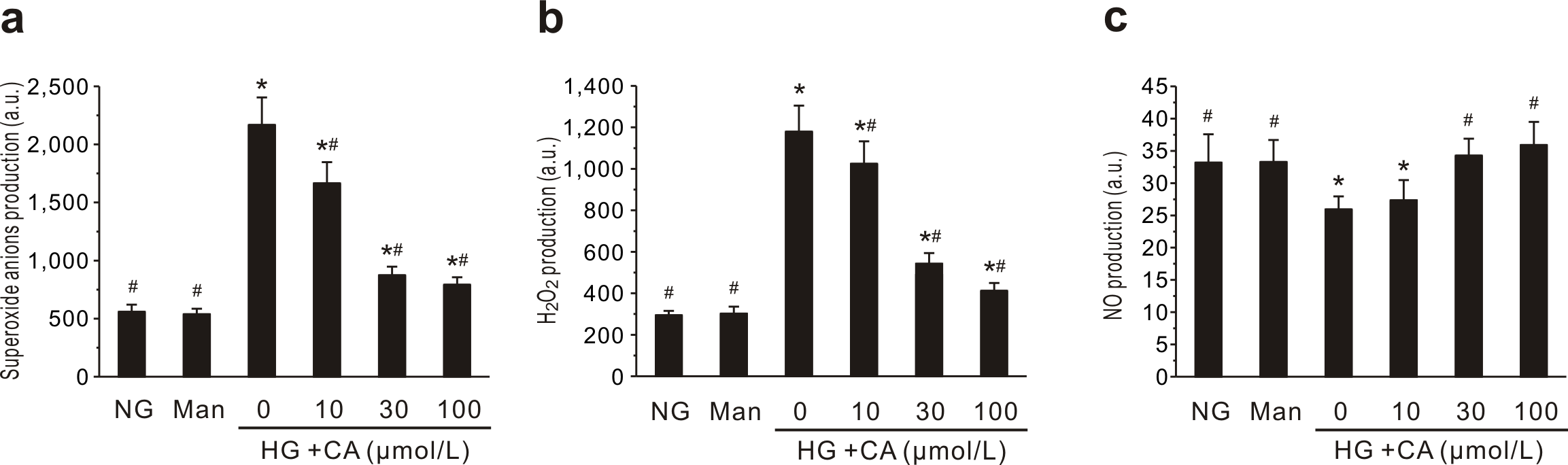


**Supplementary Fig. S3.** Effects of CA on intracellular superoxide anions (•O^2−^), H_2_O_2_ and NO. GMCs grown in serum free medium containing 5 or 25 mmol/L glucose were treated with various concentrations of CA (0-100 μmol/L) for 24 h. Mannitol (19.5 mmol/L, Man) was used as an osmotic control. Dihydroethidium (DHE), 2’,7’-dichlorofluorescein diacetate (DCF-DA) and 4,5-diaminofluorescein-2 diacetate (DAF-2/DA) were used to detect intracellular •O^2−^, H_2_O_2_ and NO using flow cytometry, respectively. Data are expressed as the geometric mean fluorescence intensity (a.u., n=4). **P*<0.05 *vs*. normal glucose (NG) control; ^#^*P*<0.05 *vs*. high glucose (HG) control.


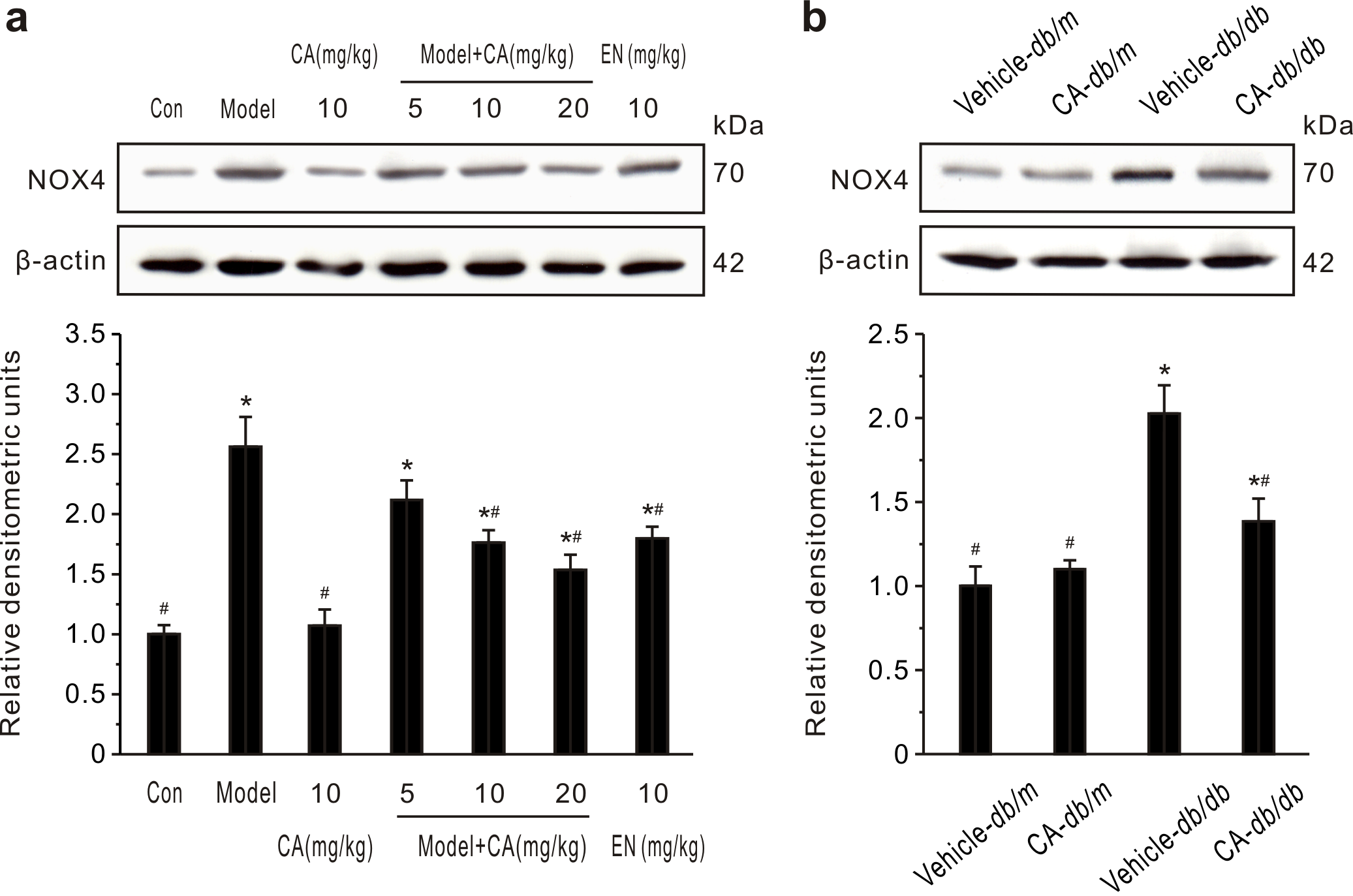


**Supplementary Fig. S4.** Effect of CA on NOX4 expression in renal cortex. *A*: NOX4 expression in type 1 diabetic rats. CA and enalapril (EN) were administered daily by gavage to STZ-induced diabetic (model) or normal (Con) rats for 8 weeks. NOX4 levels were measured by western blot analysis. A representative western blot is depicted in *upper panel*. Densitometric quantification of NOX4 relative to β-actin is summarized in *lower panel*. **P*<0.05 *vs*. Con; ^#^*P*<0.05 *vs*. model. *B*: Effects of CA on NOX4 expression in diabetic *db/db* or *db/m* mice. The mice were treated with CA(10 mg/kg/day) or vehicle (0.5% CMC) by gavage for 8 weeks. Typical results are depicted in upper panel along with statistical analysis in lower panel (*n* = 3). **P*<0.05 *vs*. vehicle-treated *db/m* mice; ^#^*P*<0.05 *vs*. vehicle-treated *db/db* mice.
